# Verification of predicted alternatively spliced Wnt genes reveals two new splice variants (CTNNB1 and LRP5) and altered Axin-1 expression during tumour progression

**DOI:** 10.1186/1471-2164-7-148

**Published:** 2006-06-13

**Authors:** Heike Pospisil, Alexander Herrmann, Kristine Butherus, Stefan Pirson, Jens G Reich, Wolfgang Kemmner

**Affiliations:** 1 Center for Bioinformatics, University of Hamburg, Bundesstrasse 43, 20146 Hamburg, Germany; 2 Dept. of Bioinformatics, Max-Delbrück Center for Molecular Medicine, Robert-Roessle Str. 10, 13125 Berlin, Germany; 3 Clinic for Surgery and Surgical Oncology, Robert-Roessle Klinik, Charité Universitätsmedizin Berlin, Lindenberger Weg 80, 13125 Berlin, Germany; 4 Children's Hospital Medical Center, Munich University of Technology, Parzivalstr. 16, 80804 München, Germany; 5 Clinic for Surgery and Surgical Oncology, Max-Delbrueck Center, Robert-Roessle Str. 10, 13125 Berlin, Germany

## Abstract

**Background:**

Splicing processes might play a major role in carcinogenesis and tumour progression. The Wnt pathway is of crucial relevance for cancer progression. Therefore we focussed on the Wnt/β-catenin signalling pathway in order to validate the expression of sequences predicted as alternatively spliced by bioinformatic methods. Splice variants of its key molecules were selected, which may be critical components for the understanding of colorectal tumour progression and may have the potential to act as biological markers. For some of the Wnt pathway genes the existence of splice variants was either proposed (e.g. β-Catenin and CTNNB1) or described only in non-colon tissues (e.g. GSK3β) or hitherto not published (e.g. LRP5).

**Results:**

Both splice variants – normal and alternative form – of all selected Wnt pathway components were found to be expressed in cell lines as well as in samples derived from tumour, normal and healthy tissues. All splice positions corresponded totally with the bioinformatical prediction as shown by sequencing. Two hitherto not described alternative splice forms (CTNNB1 and LRP5) were detected. Although the underlying EST data used for the bioinformatic analysis suggested a tumour-specific expression neither a qualitative nor a significant quantitative difference between the expression in tumour and healthy tissues was detected. Axin-1 expression was reduced in later stages and in samples from carcinomas forming distant metastases.

**Conclusion:**

We were first to describe that splice forms of crucial genes of the Wnt-pathway are expressed in human colorectal tissue. Newly described splicefoms were found for β-Catenin, LRP5, GSK3β, Axin-1 and CtBP1. However, the predicted cancer specificity suggested by the origin of the underlying ESTs was neither qualitatively nor significant quantitatively confirmed. That let us to conclude that EST sequence data can give adequate hints for the existence of alternative splicing in tumour tissues. That no difference in the expression of these splice forms between cancerous tissues and normal mucosa was found, may indicate that the existence of different splice forms is of less significance for cancer formation as suggested by the available EST data. The currently available EST source is still insufficient to clearly deduce colon cancer specificity. More EST data from colon (tumour and healthy) is required to make reliable predictions.

## Background

Alternative splicing is an important source for complexity and diversity of eukaryotic proteomes. A couple of databases hold information of published alternatively spliced forms (e.g. ASDB [1], Alternative Splicing Database [2]). A new kind of database contains splice variants detected by sequence comparison of transcripts and ESTs. These alignment based databases compare expressed sequence tags (ESTs) either with genomic sequences [3], with UniGene clusters [4-6] or with Ensembl transcripts [7]. An important advantage of this approach is the possibility to detect undiscovered splice forms [8]. In spite of some (partially solved) problems with EST data (genomic contamination or incomplete mRNA processing; see [9]), they may reflect different environmental or developmental conditions. Therefore, ESTs are an important source for the analysis of alternative splicing which is often interpreted as a regulatory mechanism for certain stages in the life span of organisms. In addititon, alternative splicing has been found to be associated with various diseases including cancer [10].

The motivation for the study presented here was (1) to validate the expression of sequences predicted as alternatively spliced by bioinformatic methods, (2) to analyse the expression of alternatively spliced genes which are relevant for pathways involved in cancer formation in colon, and (3) to try to identify splice forms which show a deregulated expression in human carcinomas.

For our experimental studies we focussed on the Wnt/β-catenin signalling pathway, which has a central role for colorectal tumourigenesis. Deregulated activation of this pathway is also observed in hepatocellular carcinomas, melanomas and uterine and ovarian carcinomas. For instance, in about 80% of all colorectal carcinomas, the tumour suppressor gene APC of the Wnt pathway carries a mutation [11]. In general, the Wnt/β-catenin pathway plays an important role for cell proliferation, differentiation, and cell motility. Wnt proteins act by activating either the "classical" Wnt pathway or one of two non-canonical signalling cascades. Constitutive activation of the Wnt signalling pathway due to mutations in the regulator genes of the pathway (β-catenin, APC and axin) leads to an increase in the cytoplasmic level of β-catenin and subsequent transcription of Wnt target genes such as c-Myc, cyclin D1 and WISP-1. An overview of Wnt pathway components which have been found to be mutated in human tumours is presented by Thorstensen and Lothe [12]. According to these authors mutations in GSK-3β, another relevant regulator molecule, have never been reported in human cancers. This might be due to the central role of GSK-3β in pathways other than the Wnt pathway and due to the fact that mutations in this gene may be incompatible with cell viability.

By bioinformatic analysis a list of putative splice variants of relevant molecules of the Wnt pathway genes was generated. RT-PCR was then used to examine gene expression of the putative sequences in several colorectal tumour cell lines, but also in patient samples derived from colon cancer and matching normal tissue.

## Results and discussions

**Figure 1:**
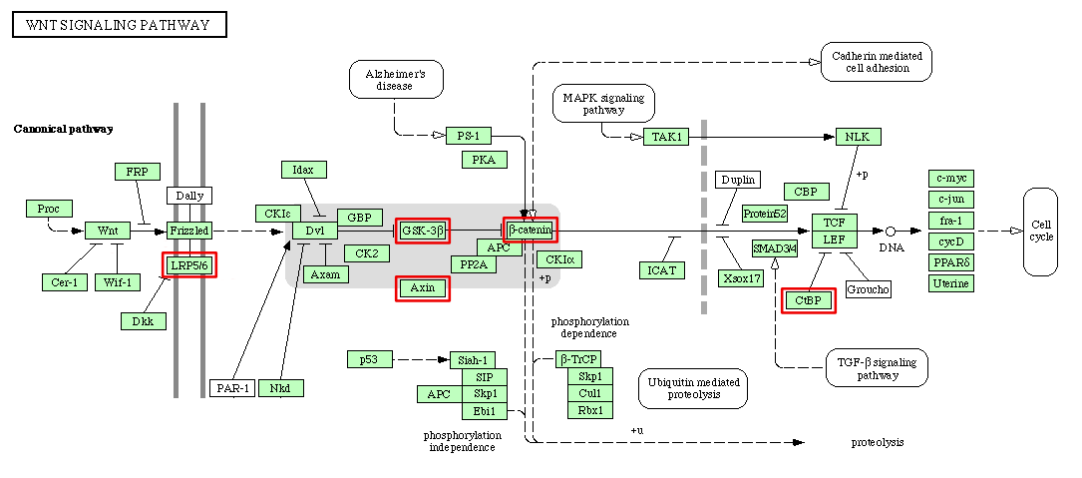
**The Wnt-pathway**. Picture taken from KEGG database . The selected genes involved in the analysis presented here are marked by red rectangles.

**Figure 2:**
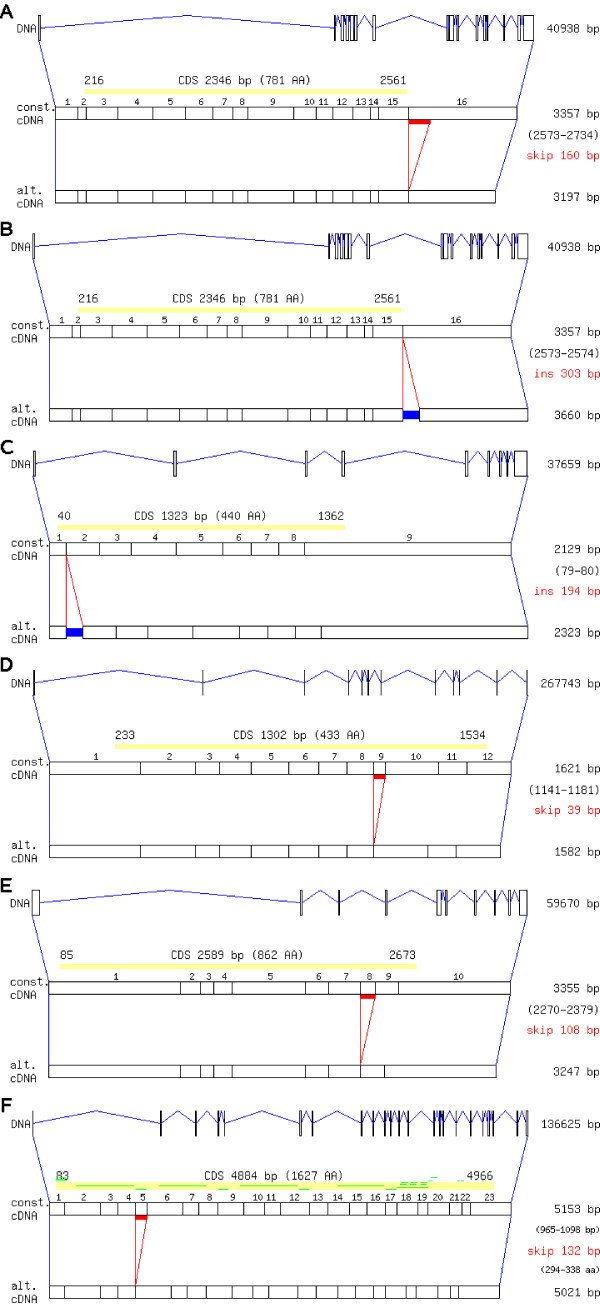
**Alternative splice forms of selected Wnt-components**. At top genomic DNA is depicted with all exons. In the middle the cDNA-structure of the normal form is given, the coding region is indicated in yellow, at the bottom the alternative isoform is shown; skips are indicated in red, inserts in blue. On the right side the length in bp is given of the pertinent sequence. For all cases, only the single splice event is shown. Potential other splice sites are neglected. A: CTNNB1(a), B: CTNNB1(b), C: CtBP1, D: GSK-3β, E: Axin-1, F: LRP5

**Figure 3:**
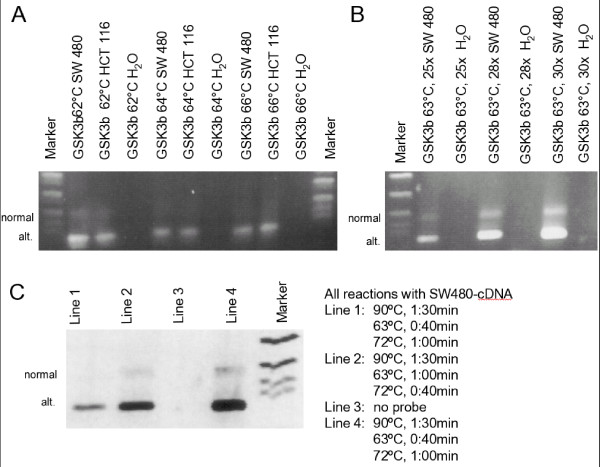
Determination of optimal RT-PCR conditions for detection of both GSK-3β splice forms. On the left (A) GSK 3β expression is shown in dependence of various annealing temperatures. On the right (B) side GSK 3β expression is shown in dependence of the number of cycles. In figure C both annealing and extension times are varied. Abbreviations: H_2_O, negative control; normal, normal GSK 3β isoform; alt, alternative splicing product.

**Figure 4:**
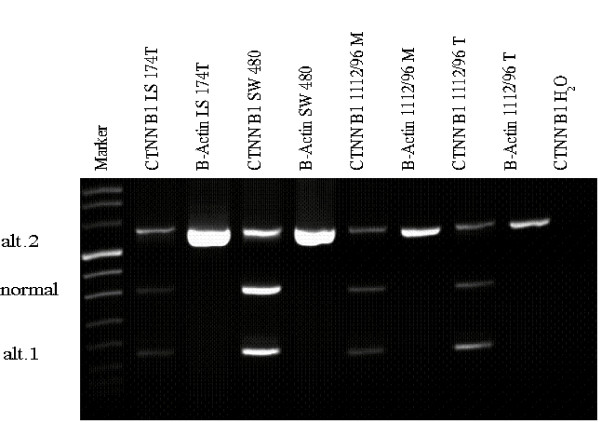
**Expression of CTNNB1 and beta-Actin**. Expression of CTNNB1 and beta-Actin in LS174T, SW-480 cell lines and tissue cDNA from colorectal mucosa and cancer. Abbreviations: H_2_O, negative control; normal, normal CTNNB1 isoform; alt.1 and alt.2, alternative splicing products.

Splicing processes might play a major role in carcinogenesis. For example CD44, Wilms tumour gene WT1, BRCA1/2 and kallikrein family genes are well known and encode cancer type related splice variants [13]. Due to the crucial relevance of the Wnt pathway for cancer progression, splice variants of the relevant molecules may be critical components for the understanding of colorectal tumour progression and may have the potential to act as biological markers. For the Wnt signalling pathway, 22 genes out of 25 are predicted to have alternatively spliced forms by EASED. Five genes involved in the Wnt signal transduction pathway were selected with respect to their role in the pathway. RT-PCR was used to examine gene expression of splice forms of β-catenin (CTNNB1), axin-1, glycogen synthase kinase (GSK-3β), C-terminal binding protein 1 (CtBP1) und low density lipoprotein receptor related protein 5 (LRP5) in several colorectal tumour cell lines, but also in patient samples derived from colon cancer and matching normal tissue. All those genes (cf. Table 3) are important key players and differ in their function and in their intracellular localization (Fig. 1). β-catenin (CTNNB1) is carrying two different splice sites. Thus, we analysed 6 EASED splice sites (Table 3). Sequence information is given in Fig. 2A–2F. We took great care in determining optimal RT-PCR conditions in order not to miss any less expressed splice forms. This is demonstrated for GSK-3β (Figure 3). Annealing temperature (3A), as well as cycling time (3B) and annealing/extension time (3C) were optimized in order to detect both splice forms. With the exception of CTNNB1, all splice sites are situated within the coding DNA and lead to changes in amino acid sequences. We used one primer set for the detection of up to three transcripts simultaneously (Figure 4). On the one hand, the presence of the different splice forms can easily compared using this procedure. On the other hand the size of the amplicon might impact the efficiency of the reaction and the expression of longer splice forms might be underestimated. As already known, the quantitative value of "hand-made" RT-PCR is limited and small expression differences will be neglected. Therefore, we used also quantitative RT-PCR (TaqMan) for one of the genes (Axin-1).

The PCR results for the 6 selected splice variants give strong evidence that the algorithm used to predict potential alternative splice forms is very reliable. The selected Wnt pathway was used as a model to test the splice variant prediction algorithm. In summary, we found that always either (1) both putative splice forms or (2) only the normal (longer) isoform are expressed in tumour and in normal mucosa. Although the underlying EST data used for the bioinformatic analysis suggested an exclusive expression in tumours neither a qualitative nor a significant quantitative difference between the expression in tumour and healthy tissues was detected. However, this result is in good agreement with Gupta et al. [14] who described that predicted tumour-specific expression of isoforms derived from ESTs usually tends not to reflect the experimentally validated expression pattern.

### β-catenin

Accumulation of β-catenin, a key molecule of the Wnt pathway, in the nucleus and interaction with TCF/LEF transcription factors leads to transcription activation of a number of target genes such as c-Myc, cyclin D1, fra1, c-Jun, matrilysin, CD44, urokinase-type plasminogen activator receptor and others [15]. These molecules are clearly relevant for tumour formation, because of their roles in proliferation, apoptosis and cell-cycle regulation. High concentration of cytoplasmic β-catenin is critical for growth of colorectal tumour cells [16]. Furthermore, the results of Hlubek et al. [17] suggest that nuclear β-catenin activates the coordinated expression of the interacting proinvasive proteins laminin gamma 2 chain and MT1-MMP, thereby leading to a promigratory activity at the invasive front of colorectal cancers. Besides its role for tumour formation β-catenin is relevant for cell-cell adhesion in conjunction with E-cadherin [18]. The existence of an additional alternative form of CTNNB1 was proposed by Nollet et al. [19]. We found this second and hitherto not described alternatively spliced isoform (Fig. 2B and Fig. 4). The sequence of this splice variant is registered under GenBank accession number AB062292. This isoform contains the complete intron 15 sequence as well as the complete exon 16. The splice site is not located within the coding region, but may affect regulatory functions of the RNA-sequence.

### CtBP1

CtBP1 might selectively repress epithelial cell adhesion and transcription of several pro-apoptotic genes. CtBP interacts with TCF-4, histone deacetylases and is able to decrease the expression of the endogenous Wnt targets [20]. Grooteclaes et al. report that CtBP-knockout cells were hypersensitive to apoptosis [21]. The expression of both CtBP1 splice variants was shown in colorectal tumour as well as in normal mucosa. However, the expression of the normal 63 bp isoform was lower. The alternative isoform contains an insert of 196 bp-length within exon 2. Due to a change of the start of the coding sequence the predicted protein is smaller than that of the normal isoform.

### GSK-3β

**Figure 5:**
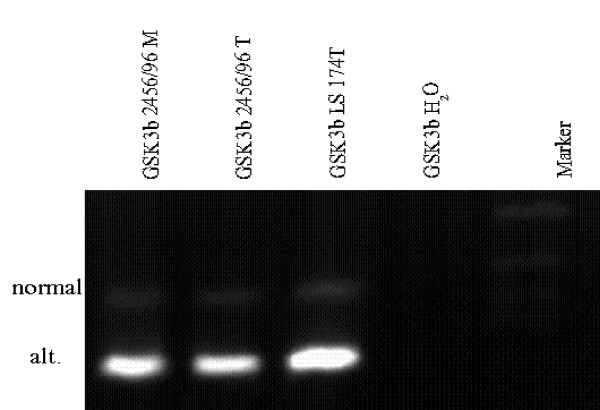
**GSK 3β expression**. GSK 3β expression in LS 174T cell line and tissue cDNA from colorectal mucosa and cancer. Abbreviations: H_2_O, negative control; normal, normal GSK 3β isoform; alt, alternative splicing product.

The serine/threonine kinase GSK-3β binds to and phosphorylates several proteins in the Wnt pathway. Phosphorylation of β-catenin by GSK-3β leads to ubiquitination of β-catenin and degradation in proteasomes [11]. No mutations of the GSK-3β gene were found in three colorectal cancer cells [22], but further studies of cells and cancer tissues are needed to test this hypothesis. The expression of both putative GSK-3β isoforms was detected in colorectal carcinomas and normal mucosa (Fig. 2D and Fig. 5). These isoforms have been annotated in Ensembl (ENST00000264235, ENST00000316626). The proteinkinase-domain (56–353 amino acids [aa]) of the alternative isoform is shorter than that of the normal isoform (13 aa between 304–316 aa) as shown before. This GSK-3β splice variant was already described by Mukai et al. [23], who localised it in human brain. Our results in colon cancer show that this splice form is not exclusively expressed in brain. Moreover, the hypothesis of Thorstensen and Lothe [12] that GSK-3α may substitute for loss of GSK-3β in tumour tissue is in agreement with our findings.

### LRP5

LDL receptor related proteins are cell surface molecules recently identified as Wnt co-receptors. It showed no effects on the canonical Wnt signalling by itself, but acted synergistically to Wnt. LRP5 binds axin and recruits axin to the membranes. Supposedly, the translocation of axin leads to stabilisation of β-catenin and target gene transcription [24]. LRP5 binds 30 or more ligands with high affinity and delivers them to lysosomal compartments, where they are degraded. Known ligands include lipoproteins, proteinases, proteinase-inhibitor complexes, matrix-proteins, bacterial toxins and viruses, and various intracellular proteins [25]. The presence of LRP5 correlates significantly with tumour metastasis in final stage osteosarcoma [26]. Fujino et al. showed that LRP5 is also required in the normal cholesterol and glucose metabolism [27]. Both LRP5 isoforms were found to be expressed in colon cancer cell lines and in patient tissues (Fig. 2F, Fig. 7). However, the expression of the alternative isoform in the tissue samples was low. Here, we describe a new, hitherto not published LRP5 isoform. Okubo et al. [28] has examined the human LRP5 gene in patients with Type III hyperlipoproteinemia by restriction enzyme fragment polymorphism (RFLP) and described seven other sequence polymorphisms, which means that a considerable heterogeneity exists in LRP5-splicing variants. On the protein level, the LRP5 exon skip would lead to the loss of part of the EGF-like -Domain (PF00008). The EGF precursor homology repeats in receptors are important for the dissociation of ligands from receptors in endocytic vesicles. The transmembrane domain is necessary for anchoring to membranes and the cytoplasmic domain is required for their targeting to coated pits and subsequent internalisation [29]. The functional relevance of this loss has to be elucidated in future studies.

### Axin-1

**Figure 6:**
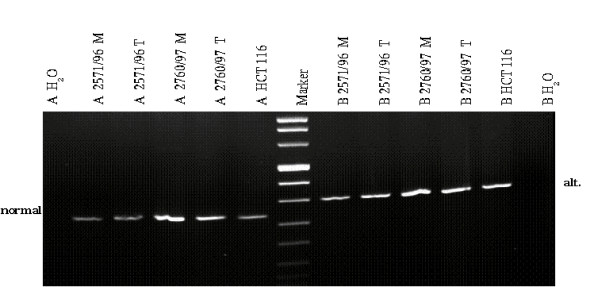
**Axin-1 expression**. Axin-1 expression in LS 174T cell line and tissue cDNA from colorectal mucosa and cancer. In the left part of figure PCR product with primer A and in the right part PCR product with primer B. Abbreviations: H_2_O, negative control; normal, normal Axin-1 isoform; alt, alternative splicing product.

**Figure 7:**
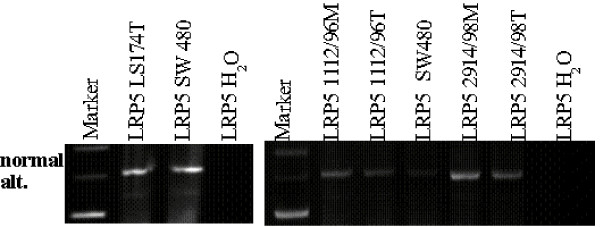
**LRP 5 expression**. LRP 5 expression in LS 174T, SW 480 cell lines and tissue cDNA from colorectal mucosa and cancer. Abbreviations: H_2_O, negative control; normal, normal LRP5 isoform; alt, alternative splicing product.

**Figure 8:**
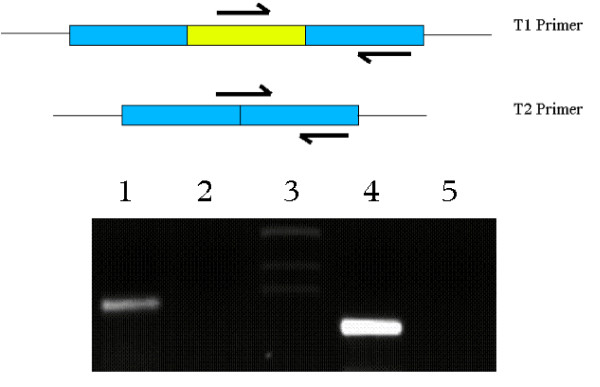
**Axin-1, primer location for real-time amplicons and electrophoresis after real-time PCR The upper part shows the primer location for real-time amplicons. T1: 104 bp amplicon (normal isoform), T2: 94 bp amplicon (alternative isoform)**. The lower part gives the electrophoresis result after real-time PCR. Line 1: LS 174S T1 primer product, line 2: negative control (H_2_O) for T1, line 3: marker, line 4: LS 174T, T2 primer product, line 5: negative control (H_2_O) for T2

**Figure 9:**
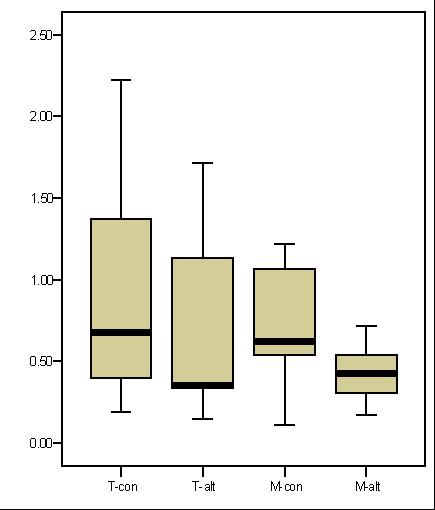
**Expression of normal and alternative axin splice forms**. Box-plots of constitutive normal (con) and alternative (alt) forms in carcinoma (T) and mucosa (M) (cases N = 14).

**Figure 10:**
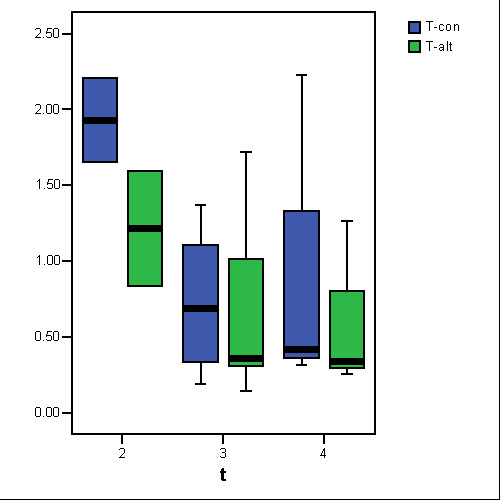
**Axin-expression in correlation with tumour invasion**. Box-plots of expression values of constitutive normal (con) and alternative (alt) forms in correlation with stage T (tumour invasion). T: carcinoma, M: mucosa; t = 2: Tumour invades muscularis propria, t = 3: Tumour invades through the muscularis propria into the subserosa, or into nonperitonealized pericolic or perirectal tissues, t = 4 : Tumour directly invades other organs or structures, and/or perforates visceral peritoneum (cases N = 14).

**Figure 11:**
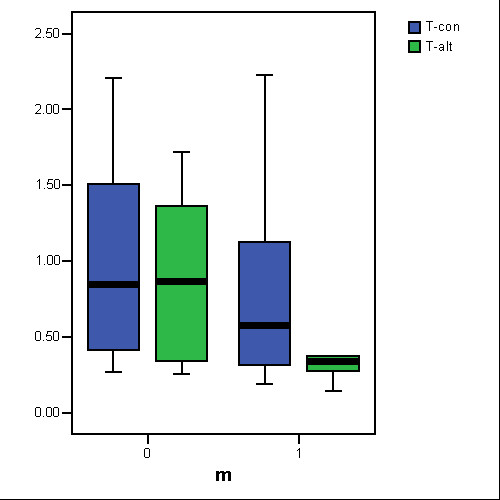
**Axin-expression in correlation with stage distant metastasis**. Box-plots of expression values of constitutive normal (con) and alternative (alt) forms of Axin in cancer tissue in correlation with stage distant metastasis. M = 0: no tumour metastasis, M = 1: distant metastasis at the time point of surgery (cases N = 14).

Axin's role as a scaffold protein in the Wnt signalling is very important for β-catenin degradation by the proteasome. So, axin acts as a negative regulator and as tumour suppressor [30]. Axin mutations were found in human hepatocarcinomas with intact genes for β-catenin and APC [31]. The expression of axin-1 was analysed by RT-PCR and quantitative real-time PCR. Exon 8 of the alternative isoform shows a 108 bp skip (Fig. 2E). The splice position was examined by RT-PCR with two different primer pairs (Fig. 6). The forward primer A (Table 1) was positioned within the skipped sequence. Therefore only the normal form is amplified by RT-PCR using this primer (Fig. 6, left part). Our results show that in RT-PCR with Axin-primer B (Fig. 6, right part) only the shorter alternative product was detected using different PCR conditions. We suppose that the PCR reaction in this case prefers the amplification of the short alternative product and used RT-PCR to examine this result. Using quantitative real-time PCR, we expected that primer T1 amplifies the normal form and primer T2 the alternative form (Fig. 8 line 1 and line 4). The results of the real-time PCR and the clinical data of patients are shown in table 4, 5. Both axin-1 splice isoforms were found in the tumour tissue as well as in healthy tissue. No differences in expression of either normal or alternative isoforms between tumour and mucosa were found (Fig. 9). However, in nearly all patient samples of normal mucosa and tumour, the alternative axin isoform was less expressed than the normal splice variant (Fig. 9). Axin expression was reduced in later stages (Fig 10). Similarly, axin expression is slightly reduced in samples from carcinomas with distant metastases (Fig. 11).

On the protein level, the alternative isoform is missing 14 aa within the DIX-domain. The DIX domain is a signalling module that is conserved in axin/conductin. In addition to this well-documented function in mediating protein-protein interactions, the DIX domain has been shown to target Dvl to actin stress fibres and vesicular membranes, and is indispensable for JNK activation by axin [32]. Axin is incapable of dimerization if carrying mutations within the DIX-Domain. This leads to the loss of axin's function as a major scaffold protein for degradation of cytoplasmic β-catenin [34]. The expression of axin was reduced in later stages (Fig. 10). Similarly, the expression of axin is slightly reduced in samples from carcinomas with distant metastases (Fig. 11). Expression of axin can be correlated inversely with depth of invasion, lymph node metastasis and lymphatic invasion in esophageal squamous cell carcinoma as shown by Nakajima [33].

## Conclusion

In summary our results demonstrate the potential of bioinformatic prediction for the search of new splice sites (as shown for CTNNB1 and LRP5) and show the precision and reliability of EST based prediction of the existence of splice sites. Otherwise, the predicted cancer specificity suggested by the origin of the underlying ESTs was neither qualitatively nor significant quantitatively confirmed. That let us conclude that EST data can give adequate hints for the existence of alternative splicing in tumour tissues, which is significant information. That no difference in the expression of these splice forms between cancerous tissues and normal mucosa was found, may indicate that the existence of different splice forms is of less significance for cancer formation as previously suggested. One source of incorrect tumour specificity could be the lack of standard and quality control in annotation of the submitted ESTs in the past. Additionally, the differing experimental results from database analysis can be caused to still incomplete EST data and a bias of EST sequences towards some tissue types and pathological states. More EST data from colon cancer as well as from healthy colon is required to have a statistical power to make precise specificity predictions. Future work is necessary to reveal whether there is strict correlation between a loss of e.g. axin expression and the tumour progression or even the formation of distant metastasis.

## Methods

### Prediction of splice variants with bioinformatic methods

The *Extended Alternatively Spliced EST Database *(EASED) [7] project is establishing a comprehensive database of alternatively spliced human mRNAs. Moreover, EASED includes useful biological information and provides the possibility to search for biologically relevant data and for candidate genes for the origin of diseases.

All transcript sequences were extracted from the Ensembl [35] web site and the repetitive elements have been marked with RepeatMasker [38]. The EST sequences were taken from dbEST [36] and were formatted for BLAST. All transcripts were aligned with the ESTs using WU-Blast [37] with an eValue of 0.001. The resulting high scoring pairs (HSPs) were analysed with an in-house written Perl-script. If one EST reveals more than one HSP with one transcript the following filtering criteria were used: (i) the gap between two HSPs has to be longer than 10 bp, (ii) both HSPs have to stretch over a sequence length of more than 30 bp, (iii) the overall identity for each HSP have to be at least 90%. The alternative splice event was named '*skip*' if the gap between two HSPs was obtain within the transcript sequence. Otherwise it was termed as an '*insert*'. All ESTs showing these HSPs with the mentioned filtering criteria were named as '*alternatively spliced ESTs*'. The corresponding '*normally spliced ESTs*' show one complete HSP without any gap over the entire EST sequence length. If possible, the HSPs from different ESTs that aligned to one transcript were clustered as follows: all HSP with start and end positions referring to the same transcript position (as an uncertainty a difference of not more than 5 bp is allowed) were merged to one single splice event. The resulting splice event positions arise from the mean of all HSP start positions and the mean of all HSP end positions.

The prediction of splice variants of investigated genes is based on Ensembl release 19 (February 2004) with 31609 human transcripts and dbEST release from December 2003 with 5,427,257 human ESTs. All processed and annotated alternative splice forms are stored in a MySQL database. It allows analysing each splice site separately instead of analysing complete transcripts. Each EST was categorised concerning its EST library annotation. At the moment of writing, EASED comprises four disease classes (*cancer, normal, healthy, unknown*), four development classes (*adult, embryo, newborn, unknown*) as well as 193 tissue classes. For the web version of EASED the 193 tissue classes were merged to 23 master classes.

In the following, we will indicate the transcript referring to the Ensembl database as the '*normal form*'. In contrast, the EST based predicted isoform is named '*alternative form*'.

### Selection of genes to be analysed

In the first selection step we searched for genes playing a major role in the Wnt/β-catenin signalling pathway with a higher number of alternatively spliced ESTs in colon cancer compared to normally spliced ESTs. To increase the number of genes we further considered genes with a comparable high percentage of alternatively spliced ESTs from other gastrointestinal tissues (as e.g. stomach) or from cancer tissues apart from colon. To further increase the number of candidates we examined the ratio of cancer ESTs to the total number of annotated ESTs. Moreover, we were interested to find out unpublished splice sequences. The selection criteria for the several genes that were chosen to be analysed are given in Table 3.

### Patients and tissue samples

Fourteen consecutive cases of colorectal cancers were examined. The patients had been treated in the Clinic for Surgery and Surgical Oncology, Robert-Roessle-Klinik in Berlin, Germany. Tissue samples were collected after obtaining informed consent. Carcinoma tissues were excised carefully from tumour specimens. Non-malignant mucosa was also scraped off in a distance of over 50 mm from tumour margin. Tissue was snap-frozen in liquid nitrogen by the Tumorbank service of the Robert-Roessle-Klinik. Carcinomas were classified and characterised according to the World Health Organization's UICC (Union Internationale Contre le Cancer) system: invasiveness (pT), lymph-node metastases (pN), primary distant metastases (M), grading (G), lymphatic vessel-invasion (L), venous vessel-invasion (V), type of resection (R0-, R1- or R2) (Table 4). Seven of these patients were women and seven were men. The age range was 51–78 years, with the median age of 64.5 years. The range of follow-up was 3–105 months, with a median follow-up of 46 months. The location of the carcinomas was as follows: rectum, four patients; colon ascendens, three patients; caecum, four patients; sigma, two patients, and one patient suffered from a carcinoma in colon transversum. Seven patients had metastases at the time of surgery. In the subsequent 2–4 years after surgery, six patients have died due to their disease. Information on gender, age, stage of disease, and histopathological factors was abstracted from the clinical and pathological records. Furthermore, material obtained during coloscopy from tumour free patients was examined for control purposes.

### Cell lines

All cell lines were of human origin, colon adenocarcinoma cell lines HT-29 (ATCC: HTB-38), HCT-116 (ATCC:CCL-247), LS-174T (ATCC: CL-188), SW-480 (ATCC:CCL-228), and mammary carcinoma cells MDA-MB-435. The adenocarcinoma lines HT-29, LS-174T, MDA-MB-435, and SW-480 were grown in Dulbecco's modified Eagle's medium, HCT-116 was grown in Iscove's medium, always supplemented with 10 % FBS, 2 mM glutamine, 100 U penicillin and 0.1 mg streptomycin. Subconfluent adherent cells were harvested by a mixture of trypsin (0.05%) and EDTA (0.02%), rescued with their own medium and washed with phosphate buffered saline (PBS).

### Tissue preparation and RNA extraction for RT-PCR

Cryo-sections from colorectal and normal tissue specimens were transferred into RLT-Buffer containing β-mercaptoethanol and frozen at -80°C. Only specimens containing more than 60% epithelial cells, no Peyer's patches and no necrotic areas were further processed. RNA was isolated using QIAshredder and RNeasy Kits. Integrity of isolated mRNA was checked by β-*Actin *One-Step RT PCR, and RNA concentration was determined photometrically. About 3 μg of RNA were used for cDNA synthesis. In the presence of 4 μM random hexamer primer RNA was incubated for 10 min at 70°C, then the mixture was held on ice for another 10 min. After addition of M-MLV-Buffer, 8 U/μL M-MLV Reverse Transcriptase, 0.8 U/μL RNasin, 0.1 μg/μL BSA and 1.25 mM dNTP and 1 h incubation at 37°C, twice the volume of absolute ethanol was added. Following 30 min at -40°C, samples were spun down in a centrifuge at 15,000 g at 4°C for 20 min and washed once with 70% ethanol. Finally, cDNA was reconstituted in 50 μL DEPC water and frozen at -20°C.

### Primer design

Primers (Table 1) used for the identification of splice variants were designed so that they bind to sequences before or after the predicted splice borders or only to the spliced sequence itself. As a result we would expect two reaction products which are different in length if a splice event takes place. All primers were produced by BioTez (Berlin). The sequences for the primer pairs were drafted using the Primer Express 2.0 software (Applied Biosystems).

### RT-PCR

RT-PCR was done mainly according to Petretti et al. [39]. The optimal concentrations, temperatures and reaction times were determined experimentally for each individual PCR. The target sequences were amplified in 25 μl reaction volume, containing: cDNA, 2 μM dNTPs (Amersham-Pharmacia Bioteches), 50 μM of each primer (BioTez, Berlin), PCR-Buffer with 1.5 mM MgCl_2 _(Applied Biosystems), AmpliTaq DNA polymerase (Applied Biosystems) and DEPC-treated water (Ambion). The PCR conditions vary for each individual gene (Table 2). Reaction products obtained after 30 cycles were electrophoretically separated in 2 % agarose containing ethidium-bromide. After electrophoresis the gels were evaluated with the gel documentation system E.A.S.Y. Win32 (Herolab). All amplicons obtained were sequenced and compared with published data. For extraction of the interesting RT-PCR reaction products from the agarose gel, Qiaex II Kit (Qiagen) or Turbo Nucleic Acid Purification Kit (Geneclean) was used. Sequence determination was done by SEQLAB (Goettingen, Germany).

PCRs were carried out for each splice (normal/alternative) position with the cDNA of six cell lines (colonic: LS-174T, SW-480, HCT-116, HT-29; mammary: MDA-MB-435, MCF-7), and with RNA extracted from surgical specimens – normal mucosa and cancer tissue of 10 patients – and two samples of normal mucosa from healthy individuals which underwent a coloscopy.

### Quantitative real-time PCR for Axin-1

For the TaqMan assays, 5 μL cDNA extracted from patient samples or LS174T cells, 100 nM T1 primers (normal axin splicing form), 200 nM T2 primers (alternative axin splicing isoform) and 150 nM β-*Actin *primers were used (primer sequences cf Table 1).

After mixing with the appropriate volume of TaqMan Universal SYBR^®^GREEN PCR Master Mix, quantitative real-time PCR was run in a MicroAmp Optical 96-Well Reaction Plate using the ABI 7000 Sequence Detection System. Thermal cycle conditions were as follows: 95°C for 10 min, initially, then 40 cycles of 95°C for 15 s and 62°C for 1 min. Dissociation curves were generated after amplification. Data was collected and analysed with ABI PRISM 7000 Sequence Detection System thermal cycler and software system (Applied Biosystems) according to comparative CT Method in the User Bulletin.

In order to analyse expression data according to the ΔC_t _method [40], C_t _values were exported from the ABI Prism 7000 SDS Software into Microsoft Excel (Seattle, WA). C_t _values of a cDNA stock from the cell line LS174T were used as calibrator. Thereby gene expression in a sample under investigation is reported as a multiple of cell culture expression. This is achieved by employing the equation (1) wherein ΔC_t _stands for the difference between C_t _values of gene of interest and housekeeper β-*Actin*.

Relative Amount = 2−(ΔCtSample.−ΔCtCalibrator.)
 MathType@MTEF@5@5@+=feaafiart1ev1aaatCvAUfKttLearuWrP9MDH5MBPbIqV92AaeXatLxBI9gBaebbnrfifHhDYfgasaacH8akY=wiFfYdH8Gipec8Eeeu0xXdbba9frFj0=OqFfea0dXdd9vqai=hGuQ8kuc9pgc9s8qqaq=dirpe0xb9q8qiLsFr0=vr0=vr0dc8meaabaqaciaacaGaaeqabaqabeGadaaakeaacqaIYaGmdaahaaWcbeqaaiabgkHiTiabcIcaOiabfs5aejabdoeadjabdsha0naaBaaameaacqWGtbWucqWGHbqycqWGTbqBcqWGWbaCcqWGSbaBcqWGLbqzcqGGUaGlaeqaaSGaeyOeI0IaeuiLdqKaem4qamKaemiDaq3aaSbaaWqaaiabdoeadjabdggaHjabdYgaSjabdMgaPjabdkgaIjabdkhaYjabdggaHjabdsha0jabd+gaVjabdkhaYjabc6caUaqabaWccqGGPaqkaaaaaa@50B5@     Equation (1)

### Statistical analysis

The SPSS software package version 10 was used for all statistical calculations. The Wilcoxon-signed-rank test was applied for calculating the difference between mRNA-expression in tumour and in mucosa of cancer patients. Associations between mRNA-expression and established histopathological parameters were calculated using Mann-Whitney- and Kruskal-Wallis-tests. A significance level of p < 0.05 was used throughout the analyses.

## Authors' contributions

AH wrote the prediction software and established the database EASED. SP participated in the selection of the Wnt components. HP and JR provided guidance for the computational work. KB performed the RT-PCR experiments under the guidance of WK. All Authors read and approved the final manuscript.

## Figures and Tables

**Table 1:** Primer information (sequence, melting temperature and PCR product length) F:Forward primer, R: Reverse primer

	GenBank Nr.	Sequence	Tm [°C]	PCR products: normal/alternative isoform [bp]
CTNNB1(a, b)	NM_001904	F:GACCTCATGGATGGGCTG R:AGGCTAGGGTTTGCTAAATTCC	60.0 60.0	329/168
CtBP1	NM_001328	F:GATGGGCAGCTCGCACTT R: TTCATGATCGGAGGTCGGAC	59.0 59.0	63/257
GSK-3β	NM_002093	F:AATGAACCCAAACTACACAGAATTTAAA R:CAATTGCCTCCGGTGGAGT	59.0 59.0	135/96
Axin-1	NM_003502	F-A:GCGCCGGTGCTGCA R-A: ACCCCACAGTCAAACTCGTC F-B:GGAGGAGGAAGAAAAGAGAGCC R-B:TGCCGATGATCTTCTCCTCAA	60.0 62.0 68.0 62.0	A:264, only normal isoform B:428/286
Axin-1, real-time PCR	NM_003502	F-T1:AGCCGTGTCGGACATGGA R-T1:AAGTAGTACGCCACAACGATGCT F-T2:GCACCCTCCAAGCAGAGAGA R-T2:CGCAGAAGTAGTACGCCACAAC	59.6 58.9 59.1 58.6	T1:104 T2:94
LRP 5	NM_002335	V-A: CCCTCTACTCACCCATGGACA R-A: AGGCACAGGTGGGAGCAG V-B: CAGCACCCGGAAGATCATTGT R-B: TCGTTGATCTCGGTGTTGACC	58 58 61 60	A:103, only normal isoform B:702/571

**Table 2:** PCR conditions, thermal cycling programs

	**CTNNB1**		**Ctbp1 (1)**		**Ctbp1 (2)**		**LRP 5**	
	°C	min	°C	min	°C	min	°C	min
activation	94	3	94	3	94	3	94	3
*Steps:*								
strand separation	90	1,5	90	1,5	92	2	92	1,5
annealing	66	1	58	1	59	1	64	1
elongation	72	1	72	1,5	72	1,5	72	1,5
number of cycles	33		30		30		30	
finale elongation	72	10	72	10	72	10	72	10
								
	**GSK 3β**		**Axin-1 (A)**		**Axin-1 (B)**			

	°C	min	°C	min	°C	min		
activation	94	3	94	3	94	3		
*Steps:*								
strand separation	90	1,5	92	1,5	90	2		
annealing	63	1	63	1	65	1		
elongation	72	1	72	1,5	72	1,5		
number of cycles	30		30		30			
finale elongation	72	10	72	10	72	10		

**Table 3:** Selected alternatively spliced Wnt pathway genes. *NAE*: the number of ESTs that predict the alternative splice form; *NCE *stands for the number of ESTs confirming the normal form. The values *NAEcolcan *and *NCEcolcan *give the respective number of ESTs deriving from colon cancer. The selection criteria for the several splice sites are given in the 8^th ^column (AE: alternatively spliced ESTs, CE: constitutive normally spliced ESTs).

Gene	Identifier (RefSeq/EASED)	AS-event	*NAE/NAEcolcan*		*NCE/NCEcolcan*		*Selection criteria*
CTNNB1 (a) *Beta-catenin*	NM_001904/ENST00000301 829 splice site 4	truncated exon 16 (160 bp skipped)	34	7	47	2	*(i) higher number of NAEcolcan*
CTNNB1 (b) *Beta-catenin*	NM_001904/ENST00000301 829 splice site 5	retained intron (303 bp inserted)	6	0	18	1	*(i) 100% cancer AE (81% cancer CE)* *(ii) unpublished splice variant*
CtBP1 *C-terminal binding protein 1*	NM_001328/ENST00000336 543 splice site 2	retained intron (194 bp inserted)	32	2	23	0	*(i) higher number of NAEcolcan* *(ii) no CE from gastroint. tissues*
GSK-3β *Glycogen synthase kinase-3 beta*	NM_002093/ENST00000264 235 splice site 1	alternative exon 9 (39 bp skipped)	26	2	1	0	*(i) higher number of NAEcolcan* *(ii) 100% AE from cancer (no annotation for CE)*
Axin-1	NM_003502/ENST00000262 320 splice site 1	alternative exon 8 (108 bp skipped)	18	0	5	0	*(i) 10 AE from cancer (1 CE from cancer)* *(ii) 2 AE from stomach (0 CE from gastroint. tissues)*
LRP5 *Low-density lipoprotein receptor-related protein 5 precursor*	NM_002335/ENST00000294 304 splice site 2	alternative exon 5 (132 bp skipped)	4	0	4	0	*(i) 100% cancer AE (67% cancer CE)* *(ii) unpublished splice variant*

**Table 4:** Clinical data of patients. Downregulation (compared to th pool cDNA der LS 174T calibrator cell line) is marked in blue, whereas upregulation is shown in red (relative amounts). Tumour classification correspond to AJCC Cancer Staging Forms 6th Ed. (2003)

**Patient**	**Diagnosis: Adeno-carcinoma of**	**death**	**TNM classification**			**location of distant metastasis**	**grading** **G**	**lymphatic vessel-invasion** **L**	**venous vessel-invasion** **V**	**type of resection** **R**
								
			**T**	**N**	**M**					
**1943**	Rectosigmoid	no	2	0	0		2	0	0	0
**1626**	Colon ascendens	no	3b	0	0		2	0	1	0
**2184**	Caecum	no	3c	0	0		2	0	1	0
**214**	Colon ascendens	no	3b	0	0		2	0	0	0
**2456**	Caecum	no	3	0	0		2	0	0	0
**2887**	Colon sigmoideum	no	4a	0	0		2	0	0	0
**2914**	Rectum	no	4b	2	0		2	0	1	0
**398**	Rectum	no	3	3	1	liver	3	1	1	0
**2104**	Caecum	yes	2	0	0		2	1	0	0
**2571**	Colon ascendens	yes	3d	2	1	liver, peritoneum	2	1	0	2
**1112**	Rectum	yes	3b	3	1	liver, lungs	2	1	0	0
**2760**	Colon sigmoideum	yes	4	2	1	liver, lungs, peritoneum	2	0	0	0
**2886**	Caecum	yes	3c	2	1	liver, kidney	3	1	1	0
**3032**	Colon transversum	yes	4	1	1	peritoneum	3	1	0	0

**Table 5:** Axin-1 quantitative real-time PCR results. The relative amounts of the quantitative RT-PCR of axin-1 for the patients listed in Table 4. Downregulation and upregulation (marked with bold font) was campared to the pooled cDNA of the LS 174T calibrator cell line.

**Patient**	**Axin RT-PCR: relative amount**			
	
	**normal mucosa**		**tumour**	
	**normal**	**alt.**	**normal**	**alt.**
**1943**	0.62	0.56	**1.65**	**1.59**
**1626**	**1.09**	0.52	0.62	**1.14**
**2184**	0.56	0.31	**1.08**	**1.72**
**214**	0.91	0.72	0.27	0.34
**2456**	**1.10**	0.54	**1.37**	0.89
**2887**	0.59	0.51	0.43	0.34
**2914**	0.39	0.26	0.40	0.26
**398**	**1.22**	0.34	**1.13**	0.34
**2104**	**1.06**	0.52	**2.21**	0.84
**2571**	0.54	0.35	0.40	0.28
**1112**	0.85	0.65	0.19	0.14
**2760**	0.29	0.17	**2.23**	**1.26**
**2886**	0.63	0.34	0.76	0.38
**3032**	0.11	0.17	0.32	0.34

## References

[B1] Dralyuk I, Brudno M, Gelfand MS, Zorn M, Dubchak I (2000). ASDB: database of alternatively spliced genes. Nucleic Acids Res.

[B2] Thanaraj TA, Stamm S, Clark F, Riethoven JJ, Le Texier V, Muilu J (2004). ASD: the Alternative Splicing Database. Nucleic Acids Res.

[B3] Kan Z, Rouchka EC, Gish WR, States DJ (2001). Gene structure prediction and alternative splicing analysis using genomically aligned ESTs. Genome Res.

[B4] Xu Q, Modrek B, Lee C (2002). Genome-wide detection of tissue-specific alternative splicing in the human transcriptome. Nucleic Acids Res.

[B5] Krause A, Haas SA, Coward E, Vingron M (2002). SYSTERS, GeneNest, SpliceNest: exploring sequence space from genome to protein. Nucleic Acids Res.

[B6] Lee C, Atanelov L, Modrek B, Xing Y (2003). ASAP: the Alternative Splicing Annotation Project. Nucleic Acids Res.

[B7] Pospisil H, Herrmann A, Bortfeldt RH, Reich JG (2004). EASED: Extended Alternatively Spliced EST Database. Nucleic Acids Res.

[B8] Roberts GC, Smith CW (2002). Alternative splicing: combinatorial output from the genome. Curr Opin Chem Biol.

[B9] Modrek B, Lee C (2002). A genomic view of alternative splicing. Nat Genet.

[B10] Venables JP (2004). Aberrant and alternative splicing in cancer. Cancer Res.

[B11] Lustig B, Behrens J (2003). The Wnt signaling pathway and its role in tumor development. J Cancer Res Clin Oncol.

[B12] Thorstensen L, Lothe RA (2003). The WNT Signaling Pathway and Its Role in Human Solid Tumors. Atlas Genet Cytogenet Oncol Haematol.

[B13] Brinkman BM (2004). Splice variants as cancer biomarkers. Clin Biochem.

[B14] Gupta S, Zink D, Korn B, Vingron M, Haas SA (2004). Strengths and weaknesses of EST-based prediction of tissue-specific alternative splicing. BMC Genomics.

[B15] Kikuchi A (2003). Tumor formation by genetic mutations in the components of the Wnt signaling pathway. Cancer Sci.

[B16] Cong F, Zhang J, Pao W, Zhou P, Varmus H (2003). A protein knowndown strategy to study the function of beta-catenin in tumorgenesis. BMC Mol Bio.

[B17] Hlubek F, Spaderna S, Jung A, Kirchner T, Brabletz T (2004). Beta-catenin activates a coordinated expression of the proinvasive factors laminin-5 gamma2 chain and MT1-MMP in colorectal carcinomas. Int J Cancer.

[B18] Hulsken J, Birchmeier W, Behrens J (1994). E-cadherin and APC compete for the interaction with beta-catenin and the cytoskeleton. J Cell Biol.

[B19] Nollet F, Berx G, Molemans F, van Roy F (1996). Genomic organization of the human beta-catenin gene (CTNNB1). Genomics.

[B20] Valenta T, Lukas J, Korinek V (2003). HMG box transcription factor TCF-4's interaction with CtBP1 controls the expression of the Wnt target Axin2/Conductin in human embryonic kidney cells. Nucleic Acids Res.

[B21] Grooteclaes M, Deveraux Q, Hildebrand J, Zhang Q, Goodman RH, Frisch SM (2003). C-terminal-binding protein corepresses epithelial and proapoptotic gene expression programs. PNAS.

[B22] Sparks AB, Morin PJ, Vogelstein B, Kinzler KW (1998). Mutational analysis of the APC/beta-catenin/Tcf pathway in colorectal cancer. Cancer Res.

[B23] Mukai F, Ishiguro K, Sano Y, Fujita SC (2002). Alternative splicing isoform of tau protein kinase I/glycogen synthase kinase 3beta. J Neurochem.

[B24] Mao J, Wang J, Liu B, Pan W, Farr GH, Flynn C, Yuan H, Takada S, Kimelman D, Li l, Wu D (2001). Low-Density Lipoprotein Receptor-Related Protein-5 Binds to Axin and Regulates the Canonical Wnt Signaling Pathway. Molecular Cell.

[B25] Strickland DK, Ranganathan S (2003). Diverse role of LDL receptor-related protein in the clearance of proteases and in signalling. J Thromb Haemost.

[B26] Hoang BH, Kubo T, Healey JH, Sowers R, Mazza B, Yang R, Huvos AG, Meyers PA, Gorlick R (2004). Expression of LDL receptor-related protein 5 (LRP5) as a novel marker for disease progression in high-grade osteosarcoma. Int J Cancer.

[B27] Fujino T, Asaba H, Kang MJ, Ikeda Y, Sone H, Takada S, Kim DH, Ioka RX, Ono M, Tomoyori H, Okubo M, Murase T, Kamataki A, Yamamoto J, Magoori K, Takahashi S, Miyamoto Y, Oishi H, Nose M, Okazaki M, Usui S, Imaizumi K, Yanagisawa M, Sakai J, Yamamoto TT (2003). Low-density lipoprotein receptor-related protein 5 (LRP5) is essential for normal cholesterol metabolism and glucose-induced insulin secretion. PNAS.

[B28] Okubo M, Horinishi A, Kim DH, Yamamoto TT, Murase T (2002). Seven novel sequence variants in the human low density lipoprotein receptor related protein 5 (LRP5) gene. Hum Mut.

[B29] Hussain MM (2001). Structural, biochemical and signaling properties of the low-density lipoprotein receptor gene family. Front Biosci.

[B30] Kishida S, Yamamoto H, Ikeda S, Kishida M, Sakamoto I, Koyama S, Kikuchi A (1998). Axin, a negative regulator of the Wnt signaling pathway, directly interacts with adenomatous polyposis coli and regulates the stabilization of β-catenin. J Biol Chem.

[B31] Satoh S, Daigo Y, Furukawa Y, Kato T, Miwa N, Nishiwaki T, Kawasoe T, Ishiguro H, Fujita M, Tokino T, Sasaki Y, Imaoka S, Murata M, Shimano T, Yamaoka Y, Nakamura Y (2000). AXIN1 mutations in hepatocellular carcinomas, and growth suppression in cancer cells by virus-mediated transfer of AXIN1. Nat Genet.

[B32] Wong CK, Luo W, Deng Y, Zou H, Ye Z, Lin SC (2004). The DIX domain protein coiled-coil-DIX1 inhibits c-Jun N-terminal kinase activation by Axin and dishevelled through distinct mechanisms. J Biol Chem.

[B33] Nakajima M, Fukuchi M, Miyazaki T, Masuda N, Kato H, Kuwano H (2003). Reduced expression of Axin correlates with tumour progression of oesophageal squamous cell carcinoma. Br J Cancer.

[B34] Luo W, Zou H, Jin L, Lin S, Li Q, Ye Z, Rui H, Lin SC (2005). Axin contains three separable domains that confer intramolecular, homodimeric, and heterodimeric interactions involved in distinct functions. J Biol Chem.

[B35] Birney E, Andrews D, Bevan P, Caccamo M, Cameron G, Chen Y, Clarke L, Coates G, Cox T, Cuff J, Curwen V, Cutts T, Down T, Durbin R, Eyras E, Fernandez-Suarez XM, Gane P, Gibbins B, Gilbert J, Hammond M, Hotz H, Iyer V, Kahari A, Jekosch K, Kasprzyk A, Keefe D, Keenan S, Lehvaslaiho H, McVicker G, Melsopp C, Meidl P, Mongin E, Pettett R, Potter S, Proctor G, Rae M, Searle S, Slater G, Smedley D, Smith J, Spooner W, Stabenau A, Stalker J, Storey R, Ureta-Vidal A, Woodwark C, Clamp M, Hubbard T (2004). Ensembl 2004. Nucleic Acids Res.

[B36] Benson DA, Karsch-Mizrachi I, Lipman DJ, Ostell J, Wheeler DL (2004). GenBank: update. Nucl Acids Res.

[B37] Gish W (1996). http://blast.wustl.edu.

[B38] Smit AFA, Hubley R, Green P (2004). RepeatMasker Open-3.0. http://www.repeatmasker.org.

[B39] Petretti T, Kemmner W, Schulze B, Schlag PM (2000). Altered mRNA expression of glycosyltransferases in human colorectal carcinomas and liver metastases. GUT.

[B40] Livak KJ, Schmittgen TD (2001). Analysis of relative gene expression data using real-time quantitative PCR and the 2(-Delta Delta C(T)) Method. Methods.

